# Exploring the Synergistic Anticancer Potential of Benzofuran–Oxadiazoles and Triazoles: Improved Ultrasound- and Microwave-Assisted Synthesis, Molecular Docking, Hemolytic, Thrombolytic and Anticancer Evaluation of Furan-Based Molecules

**DOI:** 10.3390/molecules27031023

**Published:** 2022-02-02

**Authors:** Ali Irfan, Sadia Faiz, Azhar Rasul, Rehman Zafar, Ameer Fawad Zahoor, Katarzyna Kotwica-Mojzych, Mariusz Mojzych

**Affiliations:** 1 Department of Chemistry, Government College University Faisalabad, Faisalabad 38000, Pakistan; raialiirfan@gmail.com (A.I.); sadia.faiz01@gmail.com (S.F.); 2 Department of Zoology, Government College University Faisalabad, Faisalabad 38000, Pakistan; drazharrasul@gmail.com; 3 Department of Pharmaceutical Chemistry, Riphah International University, Islamabad 44000, Pakistan; rehmanzafar016@gmail.com; 4 Department of Histology, Embryology and Cytophysiology, Medical University of Lublin, Radziwiłłowska 11, 20-080 Lublin, Poland; katarzynakotwicamojzych@umlub.pl; 5 Department of Chemistry, Siedlce University of Natural Sciences and Humanities, 3-Go Maja 54, 08-110 Siedlce, Poland

**Keywords:** benzofuran–oxadiazole, benzofuran–triazole, computational modeling, hemolytic activities, thrombolytic activities, anticancer activities

## Abstract

Ultrasound- and microwave-assisted green synthetic strategies were applied to furnish benzofuran–oxadiazole **5a**–**g** and benzofuran–triazole **7a**–**h** derivatives in good to excellent yields (60–96%), in comparison with conventional methods (36–80% yield). These synthesized derivatives were screened for hemolysis, thrombolysis and anticancer therapeutic potential against an A549 lung cancer cell line using an MTT assay. Derivatives **7b** (0.1%) and **5e** (0.5%) showed the least toxicity against RBCs. Hybrid **7f** showed excellent thrombolysis activity (61.4%) when compared against reference ABTS. The highest anticancer activity was displayed by the **5d** structural hybridwith cell viability 27.49 ± 1.90 and IC_50_ 6.3 ± 0.7 μM values, which were considerably lower than the reference drug crizotinib (IC_50_ 8.54 ± 0.84 μM). Conformational analysis revealed the spatial arrangement of compound **5d**, which demonstrated its significant potency in comparison with crizotinib; therefore, scaffold **5d** would be a promising anticancer agent on the basis of cytotoxicity studies, as well as in silico modeling studies.

## 1. Introduction

**Figure 1:**
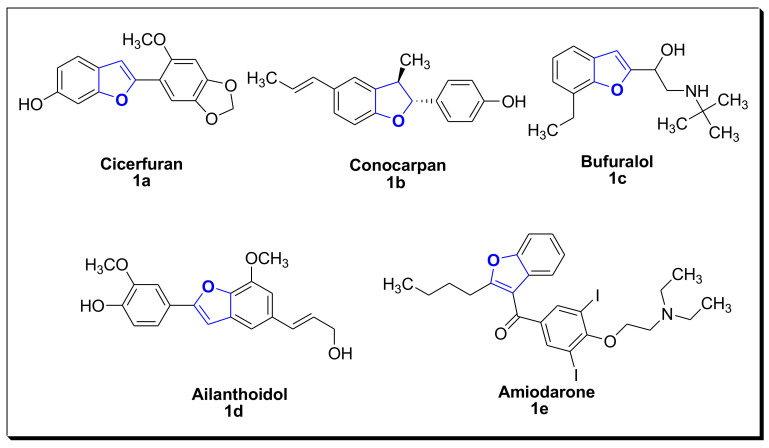
Structures of natural and synthetic furan derivatives.

In recent years, natural- and synthetic furan-based (Figure 1) based chemotherapeutic agents have attracted the attention of medicinal chemists to develop novel pharmacological agents with diverse pharmacophores andhave expanded the scope and treatment of various diseases [1,2,3].

Heterocyclic benzofurans are the basic structural units of biologically active families of natural products such as egonol, homoegonol and moracin [4]. Natural and synthetic furan derivatives exhibit profound physiological and chemotherapeutic potential against a wide variety of pathogens by displaying antimicrobial, antioxidant [5], antitubulin [6], anti-inflammatory [7], antiviral [8], antihyperglycemic [9], analgesic [10,11], anticancer [11,12], antifungal [13] and antipyretic activities [14]. The therapeutic profile of furan-based molecules has attracted medicinal researchers to design and develop anticancer agents which could play a pivotal role in cancer therapy [1,15,16]. In addition to this, oxadiazole and triazole ring systems, which are part of many natural products, have become the focus of pharmacologists and medicinal chemists on account of their medicinal and pharmacology activities, especially in the field of oncology [17,18].

**Figure 2:**
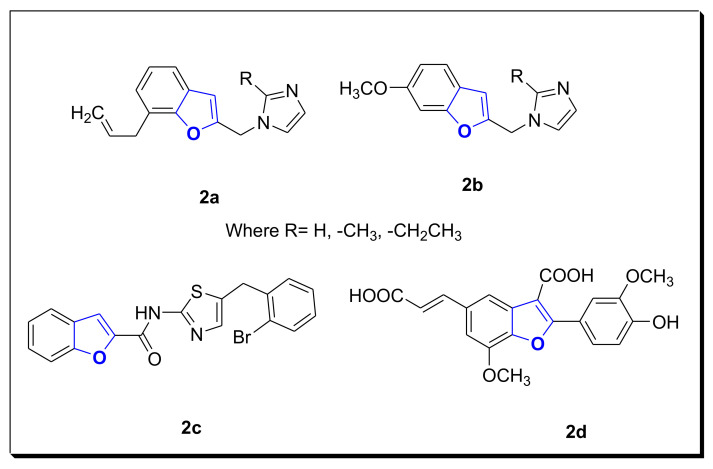
Structures of some synthetic anticancer benzofuran derivatives.

Cancer is a notably complex, prominent and lethal disease which poses a serious human health problem; ithasresulted in 7.6 million deaths globally, and this number is expected to reach 13 million by 2030 [19,20,21]. Naturally occurring benzofuran derivatives such as denthyrsin and HIBE (1-(6-hydroxy-2-isopropenyl-1-benzofuran-5-yl)-1-ethanone) displayed cytotoxic potential against ovarian cancer and human breast cancer cell lines [22,23]. Sulfonamide scaffolds of benzofuran–imidazopyridines showed anticancer activity against ovarian cancer, MCF-7, lung cancer and colon cancer [24]. Benzofuran–oxadiazole hybrids exhibited significant cytotoxicity against pancreatic cancer and colon cancer cell lines in vitro [25]. The 2-aryl benzo furan–appended 4-aminoquinazoline structural motifs significantly inhibited the epidermal growth factor receptortyrosine kinase phosphorylation in vitro against human lung cancer, cervical cancer, colorectal adenocarcinoma and hepatocellular carcinoma cell lines [26]. The benzofuran–carboxylic acid hybrids proved to be the best antiproliferative agents against breast cancer cell lines and significant inhibitors of carbonic anhydrases [27]. Thiazolodin-4-one benzofuran derivatives exhibited remarkable antitumor activity against human HEPG2 cell lines [28]. Different furan-based anticancer agents are listed in Figure 2 [1].

Continuing our previous research work towards the synthesis of benzofuran-based oxadiazole and triazole hybrids using a conventional approach [29], here, our group applied green synthetic strategies such as ultrasonic- and microwave-assisted synthetic approaches to achieve environmentally friendly synthesis with maximum production of benzofuran–oxadiazole and triazolestructural hybrids over shorter reaction times. The synthesized molecules were further evaluated for their hemolytic, thrombolytic and anticancer potential.

## 2. Materials and Methods

### 2.1. Chemistry

Ultrasonic irradiation and microwave-assisted experiments were performed, respectively, in an ultrasonic cleaner bath (Model 1510, 115 v, 1.9 L) with a mechanical timer and heater switch and at a frequency of 47 kHz, and with microwave apparatus (Model EA-180M) witha frequency of 2450 Hz and power consumption of 1150 watt. Analytical-grade solvents and reagents were used; these were purchased from Merck, Alfa Aesar and Sigma-Aldrich through local suppliers. The reaction progress was monitored using pre-coated silica gel plates. The synthesized scaffolds were purified using the column chromatographic technique and by the process of recrystallization in ethanol and methanol solvents.

### 2.2. General Ultrasound- and Microwave-Assisted Synthetic Protocols for Benzofuran–Oxadiazole Hybrids (***5a**–**g***) and Benzofuran–Triazole Derivatives (***7a**–**h***)

**Scheme 1:**
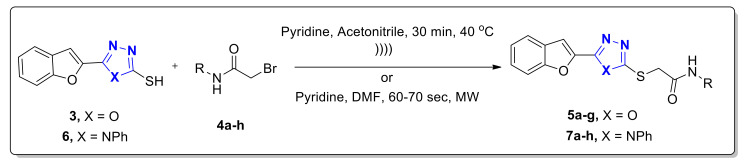
Synthetic pathway for microwave/ultrasound-assisted synthesis of *S*-alkylated oxadiazole/triazole-based benzofuran derivatives.

**Method A: Ultrasound-assisted method [30]**. The substituted *S*-alkylated oxadiazole- and triazole-based benzofuran derivatives were afforded by dissolving 5-(benzofuran-2-yl)-1,3,4-oxadiazole-2-thiol **3** (0.03 g, 0.137 mmol) and 5-(benzofuran-2-yl)-4-phenyl-4*H*-1,2,4-triazole-3-thiol **6** (0.03 g, 0.103 mmol) inacetonitrile (15 mL). Pyridine (0.213 mmol) was added, and the reaction mixture was stirred for 15 min at 0 °C. The substituted bromoacetanilides **4a**–**g** (0.24 mmol) were added, and the reaction mixture was sonicated at 40 °C for 30 min, as shown in Scheme 1. The reaction was monitored viathin-layer chromatography. On completion of the reaction, petroleum ether was added to the mixture with continuous stirring to obtain the final products in the form of precipitates, which were filtered, washed with distilled water and purified.

**Method B: Microwave-assisted method [31,32,33]**. Benzofuran–oxadiazole hybrid **3** (0.03 g, 0.137 mmol) and 5-(benzofuran-2-yl)-4-phenyl-4*H*-1,2,4-triazole-3-thiol **6** (0.03 g, 0.103 mmol) were dissolved in DMF (25 mL). Pyridine (0.213 mmol) was added to the reaction mixtureand stirred for 15 min at 0 °C. The substituted bromoacetanilide derivatives **4a**–**g** (0.24 mmol) werethen added, and the reaction mixture was irradiated in a microwave oven for 60–70s, respectively, as depicted in Scheme 1. After completion of the reaction, petroleum ether was added to the reaction mixture with continuous stirring to obtain the final products in the form of precipitates. The precipitates were filtered, washed with distilled waterand purified by recrystallization.The advantages of these preparatory protocols are simplicity, very short reaction times, generality and the elaboration of substituted benzofuran–oxadiazole and benzofuran–triazole with high to excellent yields compared toconventional synthetic approaches and microwave methods already cited for generally synthetic approaches for oxadiazole and triazole derivatives, and specifically benzofuran–oxadiazole and benzofuran–triazole scaffolds [29,34,35,36,37,38,39].

### 2.3. Biological Evaluation

Oxadiazole- and triazole-based benzofuran derivatives were evaluated for hemolysis, thrombolysis and anticancer activity.

#### 2.3.1. Hemolysis Assay

A fresh blood sample (5 mL) from a healthy donor was collected in an EDTA tube. The blood was transferred to microcentrifuge tubes and centrifugedfor 5 min at 1000 rpm to obtain the red blood cells (RBCs). The supernatant was then discarded, and the RBCpellet was washed three times with phosphate-buffered saline (PBS). The RBC pellet was collected after washing and 20µL of sample solution in DMSO was added. The tubes were incubated at 37 °C for 60 min. Having been removed from the incubator, the tubes were recentrifuged at 13,000 rpm for 5 min. The supernatant was collected and diluted with chilled PBS solution. Absorbance was recordedat 517 nm. ABTS was used as a positive control, while DMSO was employed as a negative control in this protocol. The experiment wasconducted in triplicate, and the RBC lysis percentage was calculated using this formula [40]:

$$\%\;\mathrm{Age}\;\mathrm{hemolysis}\;=\frac{\mathrm{Absorbance}\;\mathrm{of}\;\mathrm{sample}-\mathrm{Absorbance}\;\mathrm{of}\;\mathrm{negative}\;\mathrm{control}\;\times 100}{\mathrm{Absorbance}\;\mathrm{of}\;\mathrm{positive}\;\mathrm{control}}$$

#### 2.3.2. Thrombolysis Assay

The thrombolytic assay was performedaccording tomethodologyin the literature [41]. A blood sample (3 mL) was collected from a healthy human donor, and 500 µL was transferred to pre-weighed, clean Eppendorf tubes. The tubes containing the blood wereweighed again andincubatedfor 1 h at 37 °C to induce clot formation. The serum was then discarded, and the tube containing theclot was weighed. An amount of 40 µL of sample solution in DMSO was added to the clot.The tubes were again incubated for 3 h at 37 °C, and the lysis resultswere observed. ABTS was used as a positivecontrol, while DMSO was used as a negative control in this assay. The experiment was performed in triplicate, and the lysis percentage was calculated using the formula below:

$$\%\;\mathrm{Age}\;\mathrm{clot}\;\mathrm{lysis}\;=\frac{\mathrm{Initial}\;\mathrm{clot}\;\mathrm{weight}-\mathrm{Final}\;\mathrm{clot}\;\mathrm{weight}\;\times 100}{\mathrm{Initial}\;\mathrm{clot}\;\mathrm{weight}}$$

#### 2.3.3. MTT Assay

##### Preparation of Cell Culture

The human lung cancer cell line A549 was cultured in Dulbecco’s modified Eagle medium (DMEM), composed of 10% FBS (fetal bovine serum), 100 units/mL of penicillin (1%) and 100 μg/mL of streptomycin (1%), with 5% CO_2_ (carbon dioxide) at 37 °C in a moistened atmosphere. The A549 cell line was treated with synthesized compounds in a final concentration of DMSO (dimethyl sulfoxide) of less than 1%.

##### Determination of Cell Viability

The cytotoxic therapeutic potential of synthesized structural hybrids was evaluated usinga standard MTT (3-(4,5-dimethylthiazol-2-yl)-2,5-diphenyltetrazolium bromide) assay. The cells of theA549 cell line were grown, overnight, in a microculture of 96-well plates. The A549 cells were supplemented with different concentrations of compounds for 48 h and further incubated with 20 μL of MTT mixture (5 mg/mL) for 4 h at 37 °C. Following this, 150 μL of control DMSO was added to formazan crystals.The percentage of cell viability was determined viaquantified absorbance in a microplate readerat 490 nm wavelength [42].

#### 2.3.4. Molecular Docking Studies

**Figure 3:**
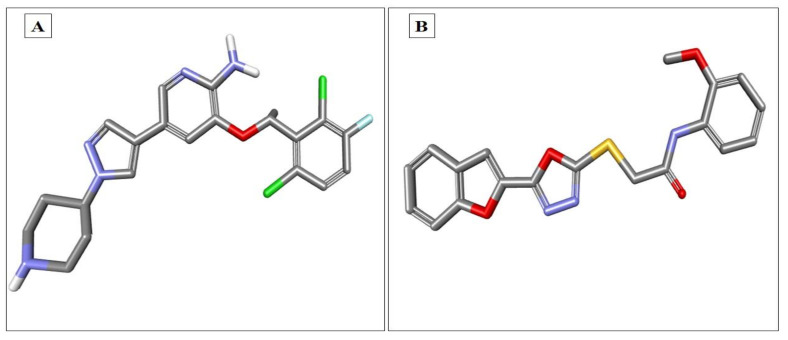
(**A**) 3D model of **crizotinib** with carbons (gray), oxygen (red), nitrogen (blue), fluorine (sky blue) and chlorine (green) shown; (**B**) 3D model of **compound 5d** with carbons (gray), oxygen (red), sulfur (yellow), nitrogen (blue) shown.

The compound **5d** with a higher pharmacological therapeutic potential was subjected to in silico studies to delineate the mechanism of anticancer activity against lung cancer A549cell lines. The molecular docking studies of these synthesized compounds at a targeted protein were carried out using auto dock Vina 1.1.2 software intermitted with PyRx. The runs of docking were achieved with grid box coordinates of −7.65, −28.27 and 43.34 for x, y and z, respectively. Considering the important role of signaling parameters in cancer, receptors of ALK (anaplastic lymphoma kinase) present an established target to evaluate the anticancer therapeutic potential of synthesized compounds [43,44]. To accomplish computational studies against this target, the crystallographic structure of anaplastic lymphoma kinase in conjunctionwith crizotinib having PDB ID: 2XP2 was obtained from the RCSB protein data bank; it was saved in Pdb formatafter the removal of water molecules and the co-crystallization ofthe ligand with the addition of polar hydrogen. The structure of the co-crystallized ligand **crizotinib** has been nominated as a potential therapeutic moiety against ALK targets;the structure of the synthesized compound **5d** was selected because of its marked experimental results. The structures were drawn on Chemdraw 20 professional software (chemoffice) and saved as a mol file [45,46]. Following this, energy minimization and polar hydrogen addition were performed, and structures were saved in Pdb format through Bio via Discovery Studio Visualizer (DSV), as shown in Figure 3. The results in the form of pictures were processed through the latest versions of Pymol and Ligplot plus software.

### 2.4. Statistical Data

The statistical data analysis was performed by using prism. All the measurements were carried out in triplicate, and the results are depicted as mean ±SD.

## 3. Results and Discussions

### 3.1. Chemistry

#### 3.1.1. Ultrasound- and Microwave-Assisted Synthesis of Oxadiazole-Based Benzofuran Derivatives (**5a**–**g**)

Ultrasound- and microwave-assisted green reactions are environmentally friendly, time saving, economical and good yield methodologies, and were adopted as depicted in Scheme 1. The conventional approach of synthesizing benzofuran–oxadiazole was already reported [29] as affording compounds in moderate to good (36–80%) yields but required a longer period of time to complete the reaction. This study was initiated to increase the yield and reduce the time of synthesized derivatives by applying ultrasound- and microwave-assisted approaches to achieve benzofuran–oxadiazole derivatives. In the ultrasound- and microwave-assisted approaches, the scaffold 5-(benzofuran-2-yl)-1,3,4-oxadiazole-2-thiol **3** was coupled with substituted bromoacetanilide derivatives **4a**–**h** to synthesize corresponding *S*-alkylated products of benzofuran–oxadiazole **5a**–**g** in good to excellent yields, as depicted in Table 1. The ultrasound-assisted strategy yielded products of 60–86% in 30 min at 40 °C, compared withthe conventional method, which afforded products of 36–80% in 24 h. The microwave approach was much more efficient in that the maximum yield of products (69–94%) was obtained in the very short duration of 60 s. The benzofuran–oxadiazole derivative **5d** was achieved withthe maximum yield, while the derivative **5f** was obtained withthe least yield via ultrasonication and microwave irradiation approaches, as mentioned in Table 1. 

#### 3.1.2. Ultrasound- and Microwave-Assisted Synthesis of Triazole-Based Benzofuran Derivatives (**7a**–**h**)

Benzofuran–triazole derivatives were synthesized via green synthetic methodologies, namelyultrasound- and microwave-assisted protocols. Benzofuran–triazole derivatives (**7a**–**h**) were obtained in low to moderate (38–79%) yields in 36 h by utilizing conventional methodology [28], while the yield was increased (60–90%) in the ultrasound-assisted synthetic protocol, in which the scaffold 5-(benzofuran-2-yl)-4-phenyl-4*H*-1,2,4-triazole-3-thiol **6** was coupled with substituted bromoacetanilide derivatives **4a**–**h** to synthesize corresponding *S*-alkylated benzofuran–triazole derivatives **7a**–**h** in good to excellent yield at 40 °Cwithin 30 min. Similarly, the microwave approach was even more efficient in that the *S*-alkylated benzofuran–triazole structural motifs were obtained in maximum yield (68–96%) within the very short duration of 70 s.The benzofuran–triazole derivative **7h** was achieved in the maximum yield (90% and 96%) in ultrasound- and microwave-assisted synthetic approaches, while the derivative **7c** was obtained in the least yield(60% and 68%) in ultrasound-assisted andmicrowaveirradiation approaches, as shown in Table 2. 

### 3.2. Hemolytic Activity

Benzofuran–triazole derivative **7b** exhibited the least cytotoxicity (0.1%) among all the synthesized derivatives, whereas benzofuran–triazole derivative **7g** (23.4%) and benzofuran–oxadiazole **5b** (22.12%) showed the highest toxicity, as depicted in Table 3. Benzofuran-oxadiazole **5d** (5.02%), **5g** (4.86%) andbenzofuran–triazole scaffolds such as **7a**, **7d** and **7f** displayed moderate results (6.13–15.7%). Benzofuran-oxadiazole hybrids **5a**, **5c**, **5e** and **5f** showed 0.5–3.7% hemolysis. The data from Table 3 also demonstrate thatbenzofuran–triazole derivatives **7b** (0.1%), **7c** (2.15%) and **7e** (3.11%) showed the least cytotoxicity among all the benzofuran–triazole derivatives against the positive control. The results demonstrate that the nature and position of the functional groups have a significant effect on the hemolytic potential of synthesized derivatives.

### 3.3. Thrombolytic Activity

All the synthesized analogues were evaluated for thrombolytic potential. Most of the benzofuran–oxadiazole and benzofuran–triazole derivatives displayed a mild to moderate thrombolytic effect when compared with the positive control ABTS. The benzofuran-triazole derivative **7f** showed the highest and best thrombolysis (61.4%) among all the synthesized hybrids when compared against the reference ABTS. The benzofuran–oxadiazole derivative **5a** exhibited significantly good lysis activity (56.8%) among all the benzofuran–oxadiazole scaffolds. The benzofuran–oxadiazole derivative **5g** and benzofuran–triazole derivative **7h** showed the least lysis (48.3% and 48.1%), respectively. Other derivatives such as **5b** (50.7%), **5c** (52.8%), **5d** (53.5%), **5e** (52.4%), **5f** (56.5%), **7a** (52.2%), **7b** (52.5%), **7c** (54.0%), **7d** (56.2%), **7e** (59.1%) and **7g** (49.06%) showed a mild thrombolytic effect, as described in Table 3. Furthermore, it wasfound that functional groups with a negative inductive effect have a positive influence on thrombolysis.

### 3.4. Anticancer Activity

The cytotoxic perspective of all the synthesized target derivatives **5a**–**g** and **7a**–**h** was evaluated against lung cancer cell line A549 in comparison with reference standard drugs crizotinib [47,48,49,50,51,52,53,54] and cisplatin [55,56,57,58,59,60] by determining cell viability usingan MTT assay, as shown in Table 3. The best and most noteworthy cytotoxicity was displayed by benzofuran–oxadiazole hybrid **5d**, with cell viability 27.49 ± 1.90 and IC_50_ 6.3 ± 0.7 μM, which was lower than the other derivatives. The benzofuran–triazole scaffold **7h** had slightly less cytotoxic potential, with a cell viability of 29.29 ± 3.98 and IC_50_ 10.9 ± 0.94 μM compared with the derivative **5d**. The benzofuran–oxadiazole and triazole derivatives **5e**, **7d** and **7g**, with cell viabilities 34.47 ± 2.19, 39.12 ± 2.21 and 36.26 ± 0.41, respectively, displayed good inhibition potential against the A549 cancercell line. The benzofuran structural hybrids **5b**, **5c**, **5f**, **5g**, **7a**, **7c** and **7e** (cell viability = 45.99 ± 4.22, 43.7 ± 0.94, 43.67 ± 4.43, 41.45 ± 4.10, 49.8 ± 1.06, 44.52 ± 5.01 and 44.72 ± 0.84) showed moderate anticancer therapeutic potential against lung cancer cells. The target benzofuran derivatives **5a** and **7b** exhibited the least cytotoxic potential, with cell viability 64.1 ± 1.72 and 57.62 ± 4.94, respectively. The benzofuran derivative **7f** was found to be inactive against the A549 lung cancer cell line with a high value of cell viability (99.1 ± 5.04), as depicted in Table 3. 

### 3.5. Structure–Activity Relationship (SAR)

**Figure 4:**
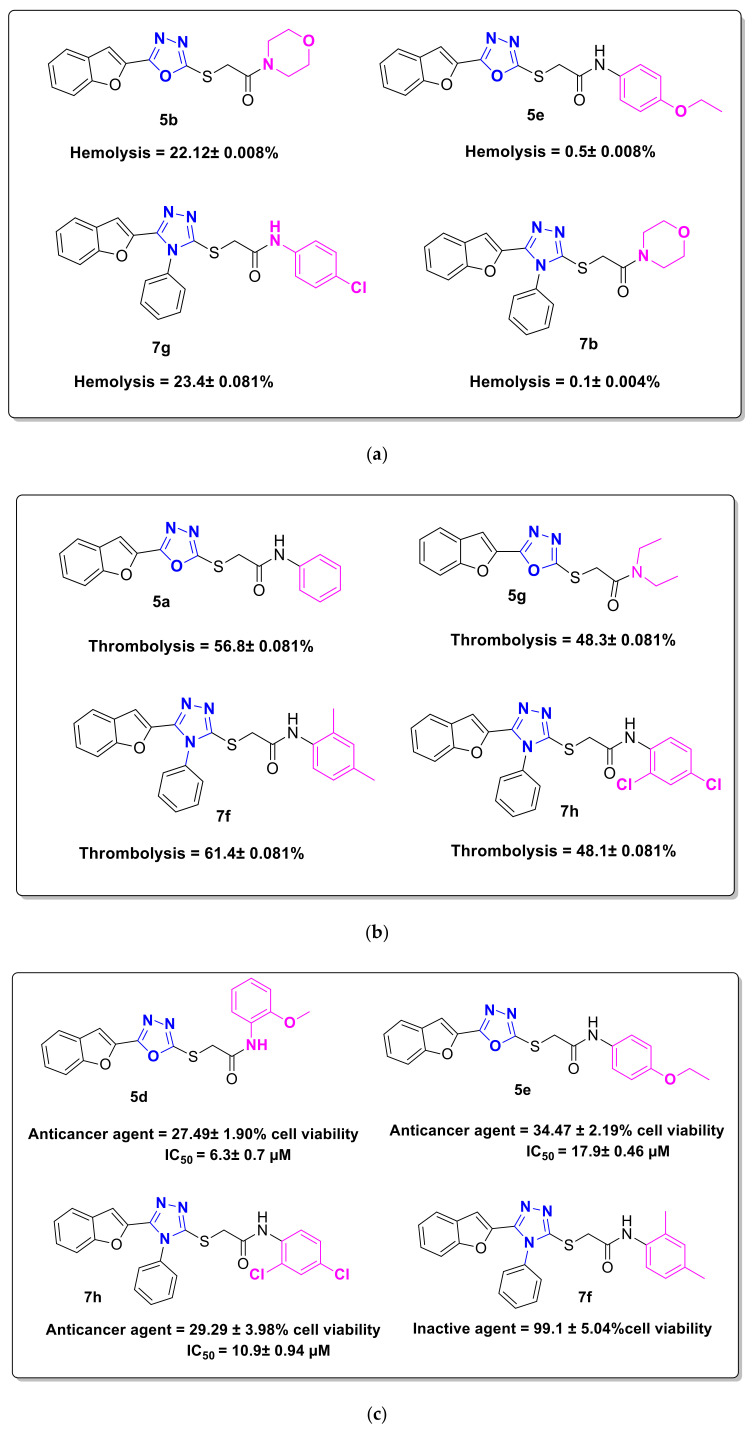
(**a**) Structure–activity relationship of hemolytic compounds **5b**, **5e**, **7g** and **7b**. (**b**) Structure–activity relationship of thrombolytic **compounds 5a**, **5g**, **7f** and **7h**. (**c**) Structure–activity relationship of anticancer **5d**, **5e**, **7h** and **7f derivatives**.

The structure–activity relationship ofhemolysis, thrombolysis and the anticancer potential of benzofuran–oxadiazole and triazole hybrids can be interpreted on the basis of substitution patterns present on the phenyl ring of *N*-(substituted-phenyl)-acetamide. The electron-donating ethoxy group atthe *para* position of the phenyl ring in benzofuran–oxadiazole compound **5e** showed 0.5% toxicity against RBCs, whereas benzofuran–triazole derivative **7b** exhibited 0.1% toxicity, which was significantly lower than the other derivatives. On the other hand, compounds **5b** and **7g** (22.12% and 23.4%) proved to be the most toxic compounds among the benzofuran–oxadiazole and benzofuran–triazole series, respectively (Figure 4a).

In the case of thrombolytic activity, the benzofuran–oxadiazole scaffold **5a** with unsubstituted phenyl displayed better thrombolytic potential (56.8%) compared withdiethyl amine moiety containing the **5g** benzofuran–oxadiazole hybrid. The phenyl group increased the thrombolytic potential of **5a,** while the diethylamine resulted in a decrease in thrombolytic activity (48.3%) of the **5g** hybrid. The benzofuran–triazole hybrid **7f** was the most active thrombolytic agent among all the screened derivatives of benzofuran–oxadiazole and benzofuran–triazole, displaying the highest thrombolysis potential (61.4%) due to the presence of two methyl groups adjacently attached on the phenyl ring at ortho *para* positions. The benzofuran derivative **7h** showed the least thrombolytic potential (48.1%) compared with all thetested compounds. The thrombolysis activity of **7h** was significantly decreased due to the presence of two chloro EWD groups adjacently attached on the phenyl ring at the *ortho* and *para* positions, as depicted in Figure 4b.

The benzofuran–oxadiazole derivative **5d** exhibited excellent anticancer activity (cell viability 27.49 ± 1.90% and IC_50_ 6.3 ± 0.7 μM) compared withall the screened derivatives, due to the presence of a methoxy group at the *meta* position on the phenyl ring;because of the presence of the ethoxy group at the *para* position on the phenyl ring, however, the benzofuran–oxadiazole derivative **5e** displayedmoderate anticancer activity (cell viability 34.47 ± 2.19% and IC_50_ 17.9 ± 0.46 μM) in comparison with the reference drugs crizotinib and cisplatin (28.22 ± 3.88 and 15.34 ± 2.98 μM), respectively. The structural hybrid **5d** is more potent than crizitonib and showed less cytotoxic potential than cisplatin. Due to the presence of two chloro EWD groups adjacently attached on the phenyl ring at the *ortho* and *para* positions, benzofuran–triazole scaffold **7h** exhibited slightly less potency (cell viability 29.29 ± 3.98% and IC_50_ 10.9 ± 0.94 μM) against lung cancer cell line A549 compared with **5d** scaffold, which was the most active compound among all the screened derivatives. The benzofuran–triazole hybrid **7f** was an inactive compound (cell viability 99.1%) against the A549 cell line due to the presence of two methyl groups adjacently attached on the phenyl ring at the *ortho* and *para* positions (Figure 4c).

### 3.6. Computational Modeling Studies of the Most Active Compound ***5d***

The synthesized compound **5d** (**B**) and co-crystallized ligand **crizotinib** were docked against the anaplastic lymphoma kinase (ALK) receptors to understand better the mechanisms of attachment and inhibition. The compound-docked conformations were interpreted by binding interactions, energies, polar bonding and ligand–receptor binding. 

The molecular comprehension of docking experimentssuggest that compound **5d** (**B**) is more prominent as it produces excellent results with binding energies of −9.925 Kcal/mol in the best binding mode. This exceeds the average energy threshold of −8.985 Kcal/mol given by crizotinib, thereby commending the improved affinity of synthesized compound **5d** (**B**) against the ALK receptor (Table 4).

**Figure 5:**
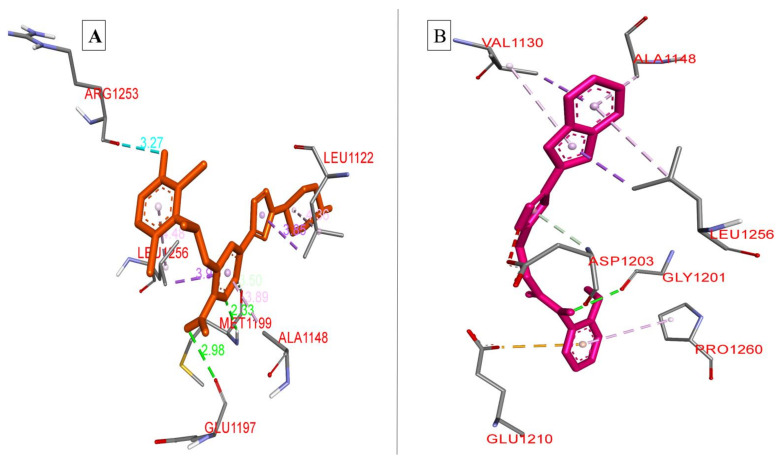
(**A**) 3D Model of **crizotinib** (brown) inside the bonding pocket of protein with interactions shown; (**B**) 3D model of compound **5d** (pink) with all atoms shown, collaborating with amino acid residues with bond distances displayed.

The active binding site of an ALK receptor prominently consists of ALA: 1252, ARG: 1253, ASN: 1254, CYS: 1255, LEU: 1256, LEU: 1257, THR: 1258, CYS: 1259 and PRO: 1260. Crizotinib was found to have noticeable contacts with these active site residues, but compound **5d** (**B**) displayed more effective binding at these active amino acid AA residues, representing more effective working of this synthesized chemical moiety. The binding of compound **5d** (**B**) and crizotinib with receptors are elaborated in Figure 5. The phenyl and heterocyclic rings of compound **5d** (**B**) showed π–sigma interaction with LEU A: 1256 and VAL A: 1130, while the NH group gave conventional hydrogen bonds with GLY A: 1201. Another prominent interaction appeared with GLU A: 1210 through π–anion linkage.

**Figure 6:**
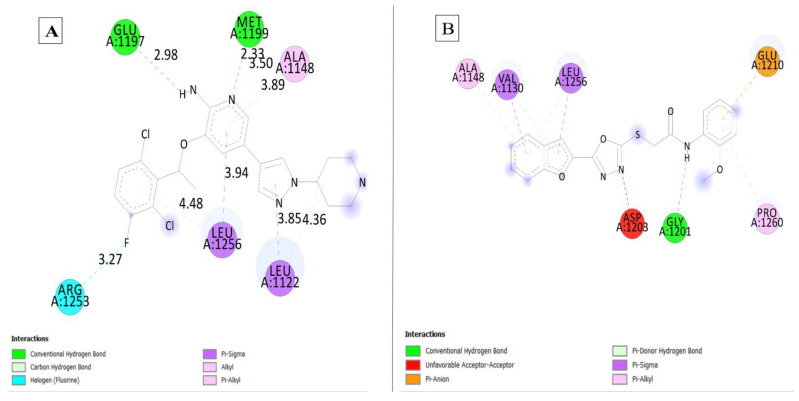
(**A**) 2D Model of **crizotinib** inside the bonding pocket of protein with interactions shown; (**B**) 2D model of compound **5d** (**B**) with all atoms shown, collaborating with amino acid residues with bond distances displayed.

Compound **5d**(**B**) was docked to determine its interaction with the subject protein and exhibited interesting results. It gave the negative binding energies of −9.925 Kcal/mol in its best binding mode. The prominent amino acid residues were VAL A: 1130, ALA A: 1148, GLY A: 1201, ASP A: 1203, GLU A: 1210, LEU A: 1256 and PRO A: 1260, as shown in Figure 6.

Evaluation through docking studies provides good evidence that synthesized compound **5d** presents excellent interaction inside the binding pocket of a receptor, showing its better anticancer potential as compared withthe co-crystallized ligand crizotinib. These computational studies are reliable and appropriate for performing binding interaction prediction. The conformational analysis revealed thespatial arrangement of compound **5d,** justifying it to be more potent, in comparison with crizotinib. Interestingly, compound **5d** interactions were more realistic, with better results. Therefore, scaffold **5d** would be a promising anticancer agent on the basis of both cytotoxicity and in silico studies.

## 4. Conclusions

The target *S*-alkylated structural motifs of benzofuran–oxadiazole and –triazole were synthesized in good to excellent yields by utilizing ultrasound- and microwave-assisted green synthetic protocols. The compounds were evaluated for anticancer, hemolytic and thrombolytic activities, and it wasrevealed that most of tested derivatives displayed significant hemolytic, thrombolytic and anticancer activities. The hemolysis assay indicated that benzofuran–triazole derivative **7b** and benzofuran–oxadiazole **5e** proved to be the least toxic compounds among all the synthesized derivatives. The benzofuran–oxadiazole hybrid **5d** with cell viability 27.49 ± 1.90 and IC_50_ (6.3 ± 0.7 μM) values was recognized as the most potent anticancer candidate against lung cancer cell line A549 compared with the reference drugs crizotinib (IC_50_ 8.54 ± 0.84 μM) and cisplatin with (IC_50_ 3.88 ± 0.76 μM). The docking studies explored the mode of action and inhibition mechanism of compound **5d** againstcancer cells; the results of the comprehensive docking analysis of compound **5d** are consistent and in agreement with biological diagnostic findings. Overall, the current studies suggest that benzofuran-linked oxadiazole and triazole hybrids are capable of being established as lead compounds in cancer therapy; in particular, scaffold **5d** could be a promising anticancer agent on the basis of cytotoxicity andin silico studies. The SAR studies suggested that more modifications to oxadiazole and triazole derivatives of benzofuran may lead to advanced anticancer candidates in cancer therapy.

## Figures and Tables

**Table 1:** Yields of benzofuran–oxadiazole derivatives via conventional, ultrasound- and microwave-assisted synthetic approaches.

Compounds	R-NH	Products Percentage Yields			m.p. (°C)	
		Conventional Approach (Faiz S. et al.) [29]	Ultrasound-Assisted Approach (This Work)	Microwave-Assisted Approach (This Work)	Found	Reported (Faiz S. et al.) [29]
**5a**	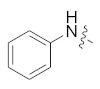	63	70	75	212	212–214
**5b**	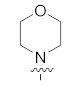	53	66	70	181–182	180–182
**5c**	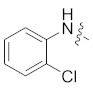	59	68	75	171–173	171–173
**5d**	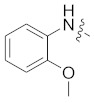	80	86	94	177	176–178
**5e**	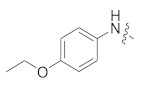	67	73	82	154–156	155–157
**5f**	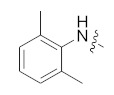	36	60	69	205	204–206
**5g**	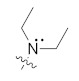	49	66	74	96–97	95–97

**Table 2:** Yields of benzofuran–triazole derivatives via conventional, ultrasoundand microwave-assisted synthetic approaches.

Compounds	R-NH	Products Percentage Yields			m.p. (°C)	
		Conventional Approach (Faiz S. et al.) [29]	Ultrasound-Assisted Approach (This Work)	Microwave-Assisted Approach (This Work)	Found	Reported (Faiz S. et al.) [29]
**7a**	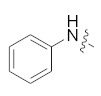	73	80	90	220–221	220–222
**7b**	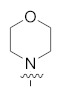	42	64	73	173–175	173–175
**7c**	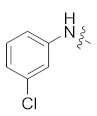	38	60	68	189–190	188–190
**7d**	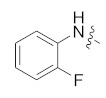	42	63	74	218–219	218–220
**7e**	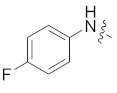	46	69	77	249–251	249–251
**7f**	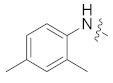	39	61	70	226–227	226–228
**7g**	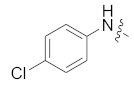	64	77	89	229–230	228–230
**7h**	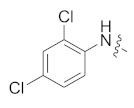	79	90	96	217–219	216–218

**Table 3:** Hemolytic, thrombolytic and anticancer activities of benzofuran–oxadiazole/triazole derivatives.

Entry	Percentage Hemolysis ± SD	Percentage Thrombolysis ± SD	^a^ Percentage Cell viability A549 (Lung Cancer) ± SD	IC_50_ (μM) A549 (Lung Cancer)
**5a**	3.7 ± 0.008	56.8 ± 0.081	64.1 ± 1.72	-
**5b**	22.12 ± 0.008	50.7 ± 0.081	45.99 ± 4.22	-
**5c**	1.3 ± 0.008	52.8 ± 0.081	43.7 ± 0.94	-
**5d**	5.02 ± 0.008	53.5 ± 0.081	27.49 ± 1.90	6.3 ± 0.7
**5e**	0.5 ± 0.008	52.4 ± 0.081	34.47 ± 2.19	17.9 ± 0.46
**5f**	0.74 ± 0.008	56.5 ± 0.81	43.67 ± 4.43	-
**5g**	4.86 ± 0.047	48.3 ± 0.081	41.45 ± 4.10	-
**7a**	9.6 ± 0.081	52.2 ± 0.081	49.8 ± 1.06	-
**7b**	0.1 ± 0.004	52.5 ± 0.081	57.62 ± 4.94	-
**7c**	2.15 ± 0.008	54 ± 0.081	44.52 ± 5.01	-
**7d**	6.13 ± 0.047	56.2 ± 0.081	39.12 ± 2.21	-
**7e**	3.11 ± 0.008	59.1 ± 0.008	44.72 ± 0.84	-
**7f**	15.7 ± 0.081	61.4 ± 0.081	99.1 ± 5.04	-
**7g**	23.4 ± 0.081	49.06 ± 0.047	36.26 ± 0.41	19.8 ± 0.54
**7h**	14.8 ± 0.081	48.1 ± 0.081	29.29 ± 3.98	10.9 ± 0.94
**ABTS** **(+ve control)**	95.9	86	-	-
**DMSO** **(-ve control)**	-	-	100 ± 0	-
**Crizotinib [41,42,43,44,45,46,47,48]**			28.22 ± 3.88	8.54 ± 0.84
**Cisplatin [49,50,51,52,53,54]**			15.34 ± 2.98	3.88 ± 0.76

^a^ Cell viability, IC_50_: (Mean ± SD) in triplicate.

**Table 4:** Molecular docking parameters analysis against ALK receptors.

Target	Ligand	Binding Energies (Kcal/mol)	Binding Residues	Type of Interaction	Bond Distance Range (Å)
Anaplastic lymphoma kinase (ALK) receptors	**Crizotinib**	−8.985	LEU A: 1122, ALA A: 1148, MET A: 1199, GLU A: 1197, ARG A: 1253, LEU A: 1256	Conventional hydrogen bond, carbon hydrogen bond, pi-sigma, alkyl interaction, pi-alkyl, van der Waals interactions	2.98–4.48
	**Compound 5d**	−9.925	VAL A: 1130, ALA A: 1148, GLY A: 1201, ASP A: 1203, GLU A: 1210, LEU A: 1256, PRO A: 1260	Conventional hydrogen bond, pi-anion, pi-donor hydrogen bond, pi-sigma, pi-alkyl, van der Waals interactions	2.68–4.57

## Data Availability

Not Applicable.

## Sample Availability

Samples of the compounds are available from the authors.
